# Supplementary material for: A unified framework for species spatial patterns: Linking the occupancy area curve, Taylor's Law, the neighborhood density function and two‐plot species turnover
Source: Ecol Lett. 2021 Aug 4;24(10):2043–53. doi: 10.1111/ele.13788 (PMC8518128; doi:10.1111/ele.13788)
Supplement: Supplementary file 4 — Supplementary Material [file ELE-24-2043-s004.zip › calcs/1-plot-data/bci-plots/annosp.pdf]

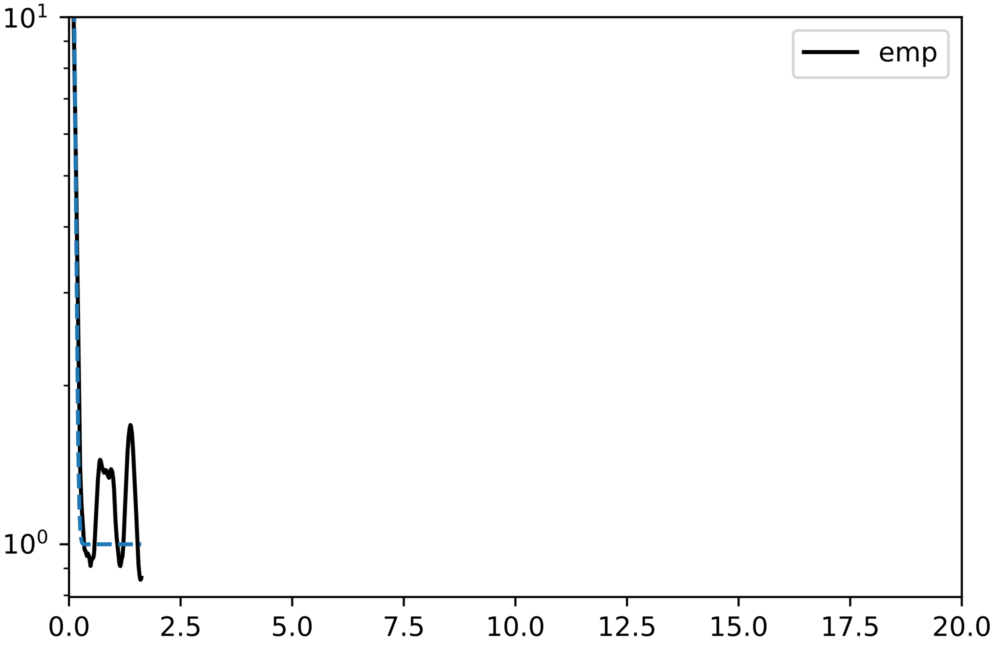

10^1
10^0
0.0
2.5
5.0
7.5
10.0
12.5
15.0
17.5
20.0
emp
